# Narratives of hospital health care professionals during the pandemic in the Northern Philippines: A phenomenological study

**DOI:** 10.4102/jamba.v14i1.1284

**Published:** 2022-09-28

**Authors:** Julius T. Capili, Lara Melissa G. Luis, Jay Emanuel L. Asuncion, Jennifer L. Luyun, Jake B. Canapi, Erwin L. Rimban

**Affiliations:** 1 College of Allied Health Sciences, Faculty of Medical Laboratory Science, Cagayan State University, Tuguegarao City, Cagayan, Philippines; 2 College of Allied Health Sciences, Faculty of Public Health, Cagayan State University, Tuguegarao City, Cagayan, Philippines

**Keywords:** hospital health care professionals (HCPs), COVID-19, coping strategies, lived experience, disaster preparedness, Philippines

## Abstract

Hospital health care professionals (HCPs) play a vital and crucial role in saving the lives of patients afflicted with the coronavirus disease 2019 (COVID-19). As the incidence of the disease keeps increasing, health care workers in hospitals face difficulties in discharging their duties. This study aimed to describe the lived experiences of HCPs. Particularly, it determined their challenges, motivations and coping strategies to craft grassroots policies for the enhancement of health care delivery in the Northern Philippines. Employing a phenomenological study design, 24 study participants were purposively selected and their narratives were elicited through focus group discussion. Responses were transcribed verbatim. After data analyses, the challenges, coping strategies and motivations of HCPs were identified. Results revealed that HCPs experienced a lot of challenges. Some of these were brought by the rapid changes in their environment as HCPs. Moreover, there was lack of comprehensive strategies that made them unprepared along with a lack of human and material resources. Furthermore, they experienced physical fatigue because of overwhelming workload, anxiety, fear and discrimination that led to depression. Despite these difficulties, they remain resilient because of religious coping, being true to their duty as HCPs and the support they receive from their fellows. Thus, the study recommends that protocols to ensue should centre on adequate pandemic preparedness and capacitation of HCPs.

## Introduction

The coronavirus disease 2019 (COVID-19) has been classified as a pandemic by the World Health Organization on 11 March 2020 (WHO 2020) considering the virus’s wide geographical spread. It affected people globally, independent of race, geography, cultural beliefs and socio-economic class (Doherty 2013). On the global scale, the COVID-19 epidemic introduced new difficulties and growing societal realities. It has radically altered people’s perceptions of themselves, their governments and the world. In particular, COVID-19 has radically changed people’s perspectives on health practices.

The COVID-19 has been dubbed the ‘pandemic of our generation’ because of the public health threat it poses. As a result, it has prompted political, fiscal, social, cultural and institutional challenges. The nation state’s health care system has been tested to its limit.

To battle and contain the spread of the virus, countries took pre-emptive steps. Many nations declared countrywide quarantines in an attempt to mitigate the unprecedented public health problem. The SARS CoV-2 virus was initially discovered in Wuhan, China. The Chinese people applauded in jubilation as they effectively resolved the country’s health crisis through intensive efforts from their health care professionals (HCPs). The successful fight against the virus seems to have been South Korea’s experience, where the virus has been successfully suppressed. On the contrary, developed nations such as Italy, the United States and France were caught off guard by the epidemic, and they continued to face several challenges. Many health professionals who were on the front lines of fighting the epidemic have been infected, severing the health care system. COVID-19-related mortality began to rise in those nations (De Los Santos & Labrague 2021). It became a concern that the health care system might collapse as the number of sick and dying individuals increased. This escalating number of individuals infected by the virus raised public awareness and even terror of COVID-19’s devastation over the globe.

As a result, the Philippine government implemented severe preventative measures such as social distancing, enhanced community quarantine and lockdown. President Rodrigo Duterte proclaimed a State of Calamity owing to the coronavirus epidemic under *Republic Act* 11332, Proclamation No. 922 series of 2020 and Proclamation No. 929. According to the Philippines Coronavirus Disease 2019 (COVID-19) Situation Report #30 of the Department of Health (DOH), there are 2.28 million cases of COVID-19 throughout the country, with 47 074 deaths. The Department of Health recorded a total of 28 112 health care workers who tested positive for COVID-19. In connection with the rising cases of COVID-19 in the Northern Philippines, the local government’s adoption of increased quarantine and the proactive engagement of hospital health care experts in the Northern Philippines became crucial.

The fight against the coronavirus has demonstrated the importance of HCPs in society. This has also given them a fresh perspective on their worth and value. They have been dubbed the ‘new heroes’ and ‘patriots’ for their contributions as important participants in the epidemic response, risking their lives and exposing themselves to contamination (Draper et al. 2008). Their duties of being medical frontline responders have been highlighted as they worked around the clock to battle the illness, care for the sick, preserve peace and order and generally bring order to areas that may otherwise have devolved into chaos (Seale et al. 2009). These HCPs are exposed to different threats, both physical and psychological threats, whilst working tirelessly to safeguard fellow residents at all hours of the day and night (WHO 2020). They were required to work long hours under duress with sometimes few resources whilst embracing the risks that came with close contact with sick patients. Much like everyone else, health care workers are subject to the illness, which can cause them to become more anxious.

Several demographic and occupational factors were significantly associated with negative mental health outcomes such as clinically relevant levels of anxiety and depression, as well as increased levels of stress across both medical and nonmedical HCPs (Lai et al. 2020; Rossi et al. 2020; Spoorthy 2020; Xing et al. 2020; Zhang et al. 2020). A recent qualitative study examining HCPs’ (specifically nurses and doctors) experiences of caring for COVID-19 patients indicated that HCPs felt a sense of duty and care towards their patients, whilst also facing a range of both physical and mental challenges of working within a completely new context amidst a pandemic. This study also highlighted the ability of HCPs to cope and adapt during these difficult times (Liu et al. 2020).

To date, there is scant literature exploring the lived experience of hospital health care workers during the COVID-19 pandemic, particularly during the period of lockdown in the Northern Philippines. Most commonly, studies focus on a larger population, employing quantitative studies on the social and psychological determinants of health and well-being in HCPs. However, naturalistic observations and other interpretative frameworks should be undertaken. The nonpositivistic paradigms are in a position to develop a deeper understanding of cultural, social, racial, linguistic and gender values by elucidating the meanings of the narratives of HCPs (Crowther 2017; Khalid et al. 2016).

Most studies regarding the outbreak often focus on medical fields like epidemiology, virology and microbiology. However, less attention has been given to the field of social sciences in understanding the role of a pandemic as a social phenomenon. Moreover, research in the social sciences would focus more on the role of structure and organisation, emphasising the health care workers’ first-hand experiences as main actors against the pandemic. A study unfolding their lived experiences through their narratives is imperative. As there is also a dearth of studies regarding the lived experiences of HCPs, this article has been conceived to address these foregoing gaps.

This research study will explore the motivations, challenges, fears, anxieties and even triumphs in combatting the coronavirus and saving the lives of infected people. Phenomenology invites the public health researcher to suspend their prejudices and biased views to interpret, describe and piece together more adequately the conditions of public health workers battling the day-to-day struggle of a coronavirus pandemic and to realise how to care more flexibly and be attuned to patients’ needs, despite the heavy toll of this coronavirus pandemic on the lives of the Filipino people.

The therapeutic health worker’s contribution to the emergence of meaning from pandemic experiences is to understand their narratives by listening and clarifying the categories of meaning that are deciphered from their narratives (Domiati et al. 2020; Dominey-Howes et al. 2015; Erfani et al. 2020; Wang et al. 2020). In this way, by clarifying their own experiences, they can see themselves more clearly. This technique provides valuable knowledge that may be applied to furthering an individual’s development. This isn’t to say that psychoanalysis and phenomenology are diametrically opposed approaches to understanding experience. Rather, they are complementary fields that can shed light on important aspects of public health research. These ‘new heroes’ may be concerned for their own safety and that of their families. They are caught in a dilemma and are battling the pandemic (Mauder et al. 2010). As a result, it is critical to investigate more about the pandemic’s emotional impacts on these healthcare personnel (Wu et al. 2009). This research project aims to investigate the different ways in which these frontline public HCPs are encountering this unprecedented public health phenomenon, highlighting their function as an ‘encounter between two views’, immersing public health workers in the world of customers and patients and the necessity to create ‘critical appraisal of the numerous frameworks in which individuals are expected to care’ (Finlay 2014). Patterns will be investigated by uncovering individual experiences and interpreting similar themes, which will be used as data to construct and develop applicable public health policies and initiatives. Ultimately, these policies and programmes will aid future HCPs in the fight against future epidemics and pandemics.

## Methodology

### Research design

The study employed a descriptive qualitative design, explicitly using Husserl’s phenomenological approach. Phenomenology suggests that social reality can be understood by people’s lived experiences (Burns & Grove 2005). Through levels of reflection, the phenomenon being investigated unveils layers of reality or meanings not seen before. It places its emphasis on understanding the different levels of psychosocial phenomena from the participant’s point of view (Welman et al. 2015), describing their ‘lived experiences’ (Ramirez 2012; Van Manen 1996). Mainly, it focuses on deducing the essence of participants’ lived experiences (Creswell & Creswell 2018), applying the epoch’s judgment or bracketing or suspending the researcher’s judgment.

### Research participants

The research used nonprobability sampling, particularly purposive sampling, in selecting the study participants, as the nature of the study did not aim to represent the whole population of HCPs, instead attempting to elicit a depth of narratives from them until data saturation was reached and the lived experiences had already been identified (Elmusharaf 2012). Twenty-four hospital HCPs in the Northern Philippines were included in the research, composed mainly of doctors, nurses, medical technologists, radiologic technologists and respiratory therapists. The study participants were chosen based on the criteria that they must have been a hospital HCP for at least one year, who had been directly involved in giving preventive treatment or assistance to patients during the COVID-19 pandemic and who had to be willing to narrate his or her experiences as an HCP through a prior and informed consent.

There were 79% female participants and 21% male participants. In terms of employment, 75% (18 of 24) occupied a permanent position whilst the rest were either contractual or casual in terms of tenure. The identified HCPs were reached through their respective agencies to solicit their voluntary participation in the study. The prior and informed consent forms were personally distributed to all and filled out by the participants to inform them of the purpose of the study and ensure the utmost confidentiality of the data gathered.

### Research procedure

Before the commencement of the data gathering, the research assistants were capacitated through protocol orientation as they went through a training workshop in conducting focus group discussions (FGDs) and thematic analyses of the results.

The interview guide that was used to elicit the participants’ responses during the FGD was pilot tested amongst hospital HCPs in one of the private hospitals in the Northern Philippines to ensure its consistency, validity and relevance. The FGD focused on determining the challenges, motivations and difficulties, fears and struggles of HCPs during the COVID-19 pandemic. During the actual conducting of the FGD, which lasted for 1 h and 30 min, the researchers ensured that the participants were comfortable, well attended and convenient. The FGDs were undertaken in a conducive room free from any disturbance and noise, prepared by the participating institution. One of the researchers facilitated the FGDs. A mobile phone and audio-recorder recorded the participants’ responses, complemented by other researchers’ note-taking. The researchers transcribed the FGD responses. The study participants were bilingual (both Filipino and English speakers). Those responses that were in Filipino were transcribed and later translated to English. Those responses in English were transcribed verbatim. After the data had been transcribed, the researchers asked the study participants to validate the tallied responses, and later their confirmation was sought. Fundamentally, the ‘epoch’ was ensured to suspend and free the researchers from biases (Husserl 1982). Moreover, self-reflexivity was also reinforced. In addition, the safety of the participants and the researchers was regarded of utmost importance; thus, the entire process of the research study followed strictly the guidelines and policies set by the Inter-Agency Task Force.

### Ethical considerations

The authors sought ethical clearance at the Region II Trauma and Medical Center in Bayombong Nueva Vizcaya, a Philippine Health Research Ethics Board (PHREB) – accredited research ethics committee (R2TMC-IRB protocol number: 2020:020).

## Results and discussion

Following data analysis in the FGDs conducted in three district hospitals in the Northern Philippines, the following significant themes were identified.

### Challenges and difficulties of hospital healthcare professionals during the COVID-19 pandemic

#### Rapid changes

Hospital HCPs experienced unforeseen changes in their lifestyles. They faced multiple immediate transitions in their working environment and practices. These changes were all so sudden that one female medical technology respondent reiterated that:

‘There is a huge adjustment in our work. We were caught off guard. We don’t have any idea on what to do and we are so helpless. We just address issues as they arise.’ (SP15, 24, Female)

Although some rapid changes were necessary to address the health and social needs of the community during the pandemic, all the research respondents agreed that these modifications on how the health care system would adjust to the new normal created a high degree of anxiety and stress aside from the fact that it doubled their workload. One nurse respondent said that:

‘The fact that we are facing a dreadful disease can bring us anxiety and fears. The stressful workload, our struggles in everyday use of PPE and the self-isolation from our families really test our emotional and mental health. Indeed, our experiences are mentally and physically draining.’ (SP6, 34, Female)

These changes became an impediment for the study participants in carrying their roles as HCPs. However, they still managed to do their best.

#### Lack of comprehensive strategy

Some respondents felt that there were no clear and comprehensive strategic plans for dealing with the pandemic. A medical doctor said that:

‘This is new to us. The health care setup was never as turbulent and shaky as this before. Whilst we are expected to be on the front row, [*we*] do not know what to do. Everyone was shocked, confused, alarmed and disturbed. The COVID-19 cases have rapidly increased. It seems there was no clear plan to end the pandemic.’ (SP8, 55, Female)

This shows the lack of preparation of the health sector in coming up with effective policies to mitigate the spread of the virus.

#### Lack of human and material resources

Availability of resources, such as personal protective equipment (PPE), rapid staff turnover and sickness of healthcare personnel, was a source of constant worry amongst the respondents. They expressed that the severe shortage of these resources made it difficult for them to discharge their duties because of the worry that they were less protected. With the shortage of essential PPE, healthcare workers were put at risk of great harm. The head nurse revealed that:

‘Sometimes we reuse our PPEs just [*so*] we feel [*more*] protected than having nothing to use at all. At the start of the pandemic, we were making our own improvised PPE, but after months, we could hardly make one because we lack time. We resort to reusing the PPE, even knowing that we can be at risk or we can be the source of the risk. It does not only refer to face masks, face shields and gloves but to many other essentials like medicines and googles. It is this time that they all suddenly become so important but very scarce.’ (SP11, 35, Male)

A resident physician seconded that:

‘In hospitals, there [*is*] too [*much*] scarcity in beds, mechanical ventilators, oxygen tanks and isolation rooms for COVID-19 positive patients. Our local response capacity has been greatly challenged, and we could hardly cope more so that incidence of diseases equally important to manage like that of COVID-19 is also fast increasing. Sometimes, we have to stop admitting patients in hospitals because we no longer can accommodate more, even when we wanted to. In fact, we just converted some more rooms to provide more space until we cannot do more except to stop accepting more patients.’ (SP20, 28, Female)

All the FGD participants aired sentiments on the shortage of manpower. It may be because of burnout and overfatigue, but the most reiterated concerns amongst the HCPs are underpayment, discrimination and the feeling of being scared because of news concerning the deaths of their colleagues.

#### Social stigma and discrimination

The pandemic has provoked social stigma and discrimination behaviours against all healthcare workers and individuals who have been in contact with the virus. Hospital HCPs also faced abusive and hurtful comments from the public. There were also several reports of false accusations of spreading the virus. The respondents felt discriminated against when they were in uniform outside duty or work. A radiology technologist commented that:

‘Many are afraid of us, including my husband. They think we are the virus. We felt humiliated and hurt when we experience such discrimination, and it is hurting.’ (SP22, 26, Female)

A nurse revealed that:

‘Some of my coworkers were banned to go back to their boarding houses, as their landlords were afraid that they carry with them the infection. Others were asked to leave, and very few were accepted but must ensure that they take a bath and make themselves sanitised before entering their dormitories or lodging.’ (SP2, 27, Female)

The social stigma and discrimination became very challenging for the HCPs to handle. This inflicted hurt on them. Whilst they were saving lives, they were being isolated and treated indifferently.

#### Physical and mental health problems

The risk of mental health problems amongst frontline healthcare workers is one of the major concerns during this pandemic. Study participants during the interview reflected on some events that occurred which challenged their mental well-being. Some claimed that they had not often talked about their feelings because their focus was on taking care of patients. Many of them explained that they had ample time to rest, which had implications for physical and mental health. Another nurse respondent stated that:

‘The fact that we are facing a dreadful disease, it can bring us anxiety and fears. It is intricate for us to gain emotional support from our families, because we are always in quarantine. The stressful workload, our struggles in everyday use of PPE and the self-isolation from our families really test our emotional and mental health. Indeed, our experiences is mentally and physically draining.’ (SP10, 35, Female)

The physical and mental well-being of the HCPs was at risk during the COVID-19 pandemic.

### Coping strategies and motivations of healthcare professionals

#### Religious coping

The Philippines is a Christian country and dominated by Roman Catholics. It makes sense that the religious coping mechanism of HCPs was one important theme from the data collected from the FGDs conducted. Study participants unanimously expressed, ‘Faith over fear. … Let God’s will be done’. A nurse administrator added that:

‘God is the reason why we are waking up every day. We just continue praying. He will give us strength to serve the people. We need to trust divine guidance and protection.’ (SP6, 34, Female)

It is through religious coping that the study participants found hope to continue and become resilient in carrying out their duty.

#### The call of duty

Some respondents claimed that stress, anxiety and fears were simply a part of their duty as HCPs. They define it as their ‘call of duty’. Respondents also stated that they accept all public criticisms, views and opinions, move forward and then do their job. A male nurse said that:

‘This is our calling. We took an oath to discharge our responsibility faithfully and excellently. Stress, exhaustion and criticisms are all part of our job – they are not new to us. People need us as much as we need them. That’s just it.’ (SP11, 35, Male)

It was clear that the primary motivator of the respondents was their responsibility to their patients.

#### Support and praise to the modern heroes

Study participants also revealed that support and praise from other people motivated them to work and serve the country. The medical doctor responded that:

‘The moment that we feel the support was there and we [*were*] regarded [*as*] heroes, it is enough consolation that makes us more motivated to work. Saving the COVID-19 patients now means learning to save the world in the future pandemics.’ (SP8, 55, Female)

A nurse said that:

‘We feel overwhelmed by the gesture of respect and kindness from people. I believe that a lot of people and churches are praying for HCPs. That is other than the food packs, medical supplies, messages they send in social media aside from some other stuff they give. It keeps me going knowing that people are also sharing something for us and that they too are concerned about us.’ (SP2, 27, Female)

The encouragement the HCPs received from various people intensified their resolve to serve their patients despite the difficult times. They experienced feelings of relief, especially when people called them modern heroes.

### Recommendations for future pandemics and outbreaks

#### Adequate pandemic response

From the arising challenges and motivational strategies of HCPs, most of them recommended that protocols to ensure safety and well-being of medical frontliners should centre on adequate pandemic preparedness. There is a need to vaccinate all employees and volunteer workers in the hospital, even those undergoing on-the-job trainings or internships, in order to avoid contracting the disease. The triage system should be assessed as to its effectiveness and its flaws should be addressed. Infection control plans must be implemented strictly as one case, even when it is isolated, can put hundreds of lives in danger. A respondent nurse reiterated the importance of this point. However, it was disclosed by a respondent in the FGD that there were cases when administrators used their influence to get slots for patients they referred. It was as if it came as their privilege:

‘There should not be any special treatment for VIPs, especially those endorsed by politicians and officials in the triage. These officials should be first in clinging to the policies.’ (SP10, 35, Female)

#### Hospital resilience

Health care systems and institutions must increase hospital resilience, the ability of the hospitals to cope after responding to a pandemic. There should be more effective recruitment strategies, live-in care management and training of new staff. Part of the plan is the allocation of funds to hire more workers and ensure that salaries are attractive to attract more HCPs. A respondent medical technologist said that:

‘With the volume of patients, tight work schedule, our colleagues with comorbidities not allowed to report and the requirement for being quarantined after straight days of duty makes us overworked and physically drained.’ (SP15, 24, Female)

A nurse respondent seconded that:

‘No matter how competent a hospital worker is, there is a limit to the physical and mental strength he can exert. Sometimes, I entertain the possibility of quitting because I feel so tired and helpless.’ (SP2, 27, Female)

A nurse respondent also shared that:

‘Our hazard pay and other incentives are not given on time. It is worst for the health workers in LGUs [*local government units*] since they do not receive hazard pay. It must be recommended that benefits for us must be standardised; better yet if it becomes across the board, because all of us in the health facilities are vulnerable.’ (SP10, 35, Female)

In the case of the district hospitals included in the FGD, the battle-cry of the health care providers was for compensation and fringe benefits. Nearly all who joined in the study revealed that they had not been receiving bonus pay for being at risk in their work. Other fringe benefits were not given to all HCPs. A midwife respondent said that:

‘We are all vulnerable since our exposure is the same, but extra benefits like the Special Risk Allowance [*SRA*] is given to a few, and it makes us feel rude and worthless.’ (SP3, 44, Female)

The hazard pay accounted for about 10% – 25% of medical personnel, but it was subject to the availability of funds from the local government unit. The Special Risk Allowance (SRA) is a benefit given by the government, but it is limited to doctors, nurses, midwives and medical technologists only.

Moreover, a common recommendation from amongst the frontliners in hospitals referred to the need to add more equipment and isolation rooms. With the hospitals congested by the number of patients, the disinfection time before more patients could be admitted after one had been discharged seemed like a hustle for the frontliners. The majority claimed that time was of the essence and that there was not much time to wait. Whilst PPE is disposable, nebulisers, mechanical ventilators, beds and other apparatuses needed to be disinfected. Whilst this was a deviation from the health protocols as prescribed by the WHO, the practice was carried out so that it would make processes and transactions faster. A respondent nurse revealed that:

‘In the medical centre, sometimes we just need to mop the floor with disinfectants instead of the usual fumigation practices. Sometimes we instruct the ambulant patients to alcoholise their own bed … we share the responsibility of ensuring sterility.’ (SP10, 35, Female)

A pharmacy respondent also said that:

‘I have been seeing patients bringing their own oxygen tanks and beds. If they are not yet accommodated in the hospital as bed capacity is exhausted, they stay inside their own cars parked in the parking lot or they may opt to stay in the hospital lobby. These made me feel having goose bumps.’ (SP4, 58, Female)

During the onset of the COVID-19 pandemic, some rooms in hospitals had been converted into COVID wards. Should cases rise to a critical number, rooms and morgues made of tents were assembled.

Frontliners also in the hospital admitted that the government should build more hospitals and create efforts to fast-track procurement processes and monitor utilisation. Health care facilities were over-saturated and there were not enough beds and rooms to accommodate all the patients. A doctor responded that:

‘I have a heavy heart every time I see a patient breathing so difficult and cries for help, and yet I feel helpless not being able to help, because there is nothing I can do; the hospital has no vacancy.’ (SP17, 27, Male)

Even district hospitals had now been made into quarantine facilities, and as COVID-19 patients were the priority, people with other ailments like stroke and vehicular accidents were denied and later would simply die. A nurse administrator admitted that:

‘There must be comprehensive planning, and the use of the government’s money through taxpayers’ money must be rationalised.’ (SP6, 34, Female)

A medical technologist responded that:

‘While PPE is one of the most important provisions that we frontliners must have, we likewise should be [*making*] plans of how we can be insured. There must be quicker procedures for procurement to guarantee sustainability of our supplies and resources. Because of the scarcity of PPE and supplies, there are instances when we reuse PPE (wash and wear). Sometimes, they don’t usually fit us so it’s either we make adjustments or just bear with the discomfort.’ (SP16, 32, Female)

Healthcare professionals were also troubled and dismayed that some people remained uncompliant with standard safety protocols. There were those in the streets not even wearing a face mask. Others were hesitant about COVID-19 vaccination. The respondents also felt that the government was lax in implementing protocols and that policing practices were not efficient. This added stress and psychological burdens to frontliners, and thus they had been calling for time off. Whilst they had been making many sacrifices to save those who were sick, the general populace remained complacent. A nurse said that:

‘People lacks discipline. They do not even cooperate. We already feel so tired and some of us are dying, yet we see lots of people in the streets not minding that it is yet unsafe to get out.’ (SP11, 35, Male)

The concept of social distancing has not yet been widely adopted. Assemblies and celebrations still happen. There are still activities that lead to crowding. A radiologic technologist claimed that:

‘It seems that we must have better strategies to implement social distancing. We give so much effort on social shielding [*that*] often I feel strained in subjecting myself for mandatory quarantine after work. It is so much of a burden for us frontliners, but we need to do it because we care about our families and the community.’ (SP5, 45, Male)

A medical doctor also said that:

‘We hope that soon, families of frontliners like us will also be prioritised in terms of vaccination, if that can be the benefit of risking our life as health workers.’ (SP12, 47, Female)

Today, the Department of Health of the Philippines has released guidelines that direct family members of health care workers be included in the A4 criteria, identifying them to be of moderate risk as they may have potential exposure to a frontliner; however, it has not yet taken effect because of the issue on vaccination scarcity.

## Discussion

The findings of the study revealed different challenges and difficulties amongst HCPs in the Northern Philippines. Some of these are because of the rapid changes that were brought by COVID-19. Consequently, it led to new emerging realities in healthcare delivery. The lifestyles of the HCPs drastically altered. Their interactions with people and how they assessed their patients became challenging for them. This affirms the study of McGlinchey et al. (2021). Health care professionals encountered changes in their working environment, experiencing challenges in their new roles. The changes were unavoidable and generated uneasiness and anxiety. Another difficulty amongst HCPs was the lack of comprehensive strategy during the COVID-19 pandemic. This is in consonance with the study of Nyashanu et al. (2020). Their findings revealed that many HCPs reported lack of preparations within the health care sector owing to nonexistent pandemic control and management protocols. This caused panic and fear amongst HCPs as pandemic preparedness is the key to the control and management of infection in the workplace and home.

Health care professionals also experienced social stigma and discrimination. The findings are in response to the study of Franklin and Gkiouleka (2021), which suggested further research on social and societal risks such as staff shortages, intersecting inequalities and financial stressors. The results of the study mirrored the realities that the HCPs faced during the pandemic, especially in a developing country like the Philippines. Health care professionals were also exposed higher levels of anxiety, depression and posttraumatic stress disorder. This is consistent with the findings of the study by Khanal et al. (2020) in Nepal, where the results revealed that mental health outcomes amongst health care workers could be caused by too much exposure to COVID-19 patients. Also, a heavy workload was a risk factor in developing mental health problems.

However, despite these challenges and difficulties that the HCPs faced, they became resilient because of their coping strategies and motivations. Religious coping became the most solicited strategy amongst the study participants. This was in line with a recent study by Munawar and Choudhry (2020), which showed many health care frontliners used religious coping to deal with challenging situations. There was also a positive relationship between the use of religious coping and physical or mental health.

A very integral part of coping amongst HCPs was reminding themselves of the call of their duty. This duty for them transcended the self in taking care of their patients. Other people’s support and praise for HCPs were integral in encouraging and motivating them. Any form of effort to show support to HCPs is essential. It plays a crucial role in maintaining the well-being of HCPs (McGlinchey et al. 2021). The study participants also added that the idea of brotherhood and togetherness amongst their coworkers made their work easier and better. A significant influence on the motivation of health care workers is appreciating, admiring, respecting and recognising the work they do (Van Der Goot et al. 2021; Windarwati et al. 2020).

The lived experiences of the HCPs suggest that there should be adequate pandemic response and hospital resilience in health care institutions in the Northern Philippines. According to the findings of the WHO (2020), a disaster plan for pandemics should be kept in place. Health care institutions around the world were not ready to cater to pandemics, and the experiences today should be instructive for the future. Disaster management plans must not be focused on barangays and local government units which are prone to catastrophic events, but rather on social and health care institutions through a Disaster Management Council alongside with the Infection Control Committee. This will ensure an adequate pandemic response.

Results from the study done by Al Thobaity and Alshammari (2020) revealed that indeed, most health care institutions in the world have issues with a critical shortage of nurses, who are the first line of defense. The issues on staffing ratios are associated with the increasing number of COVID-19 cases, deaths amongst staff (especially nurses), staff who need to be isolated either because they are suspected or confirmed COVID-19 cases and physical exhaustion.

## Conclusion

The results of this study regarding the impact of COVID-19 suggest that district hospitals and health care institutions are not adequately equipped to deal with extreme pandemics like COVID-19. Hospital HCPs experienced unforeseen challenges and struggles, such as unpreparedness, lack of funds and supplies, anxiety, depression and discrimination. Thus, protocols to ensure the safety and well-being of HCPs should centre on adequate pandemic preparedness. A coordinated approach between the government and hospital institutions and organisations must strengthen the system’s ability to manage and contain such pandemics. Also, greater efficiency in testing and isolation of affected individuals by COVID-19 would appear key in managing and preventing the spread of the pandemic.
